# Exercise Training Preserves Ischemic Preconditioning in Aged Rat Hearts by Restoring the Myocardial Polyamine Pool

**DOI:** 10.1155/2014/457429

**Published:** 2014-10-23

**Authors:** Weiwei Wang, Hao Zhang, Guo Xue, Li Zhang, Weihua Zhang, Lina Wang, Fanghao Lu, Hongzhu Li, Shuzhi Bai, Yan Lin, Yu Lou, Changqing Xu, Yajun Zhao

**Affiliations:** ^1^Department of Pathophysiology, Harbin Medical University, No. 157 Baojian Road, Nangang District, Harbin 150086, China; ^2^Department of Pathology, Heilongjiang Electric Power Hospital, Harbin 150090, China; ^3^Department of Pathophysiology, Qiqihar Medical University, Qiqihar 161006, China; ^4^Department of Cardiology, The First Clinical Hospital of Harbin Medical University, Harbin 150001, China

## Abstract

*Background*. Ischemic preconditioning (IPC) strongly protects against myocardial ischemia reperfusion (IR) injury. However, IPC protection is ineffective in aged hearts. Exercise training reduces the incidence of age-related cardiovascular disease and upregulates the ornithine decarboxylase (ODC)/polyamine pathway. The aim of this study was to investigate whether exercise can reestablish IPC protection in aged hearts and whether IPC protection is linked to restoration of the cardiac polyamine pool. *Methods*. Rats aging 3 or 18 months perform treadmill exercises with or without gradient respectively for 6 weeks. Isolated hearts and isolated cardiomyocytes were exposed to an IR and IPC protocol. *Results*. IPC induced an increase in myocardial polyamines by regulating ODC and spermidine/spermine acetyltransferase (SSAT) in young rat hearts, but IPC did not affect polyamine metabolism in aged hearts. Exercise training inhibited the loss of preconditioning protection and restored the polyamine pool by activating ODC and inhibiting SSAT in aged hearts. An ODC inhibitor, *α*-difluoromethylornithine, abolished the recovery of preconditioning protection mediated by exercise. Moreover, polyamines improved age-associated mitochondrial dysfunction *in vitro*. *Conclusion*. Exercise appears to restore preconditioning protection in aged rat hearts, possibly due to an increase in intracellular polyamines and an improvement in mitochondrial function in response to a preconditioning stimulus.

## 1. Introduction

Ischemic preconditioning (IPC) is one of the most powerful endogenous cardioprotective mechanisms [1], and it has been documented in the young heart of every species tested [2], but this response is attenuated in the aged heart [3]. Abnormalities in gene/protein expression, signal transduction cascades, and mitochondrial function have all been proposed to be involved in this attenuation [4, 5]. An age-associated reduction in cardiac mitochondrial function and an increase in the production of reactive oxygen species (ROS) are major factors that contribute to the diminished responsiveness of the aging heart to IPC stimuli [6, 7]. However, the exact biological mechanism of this process is not yet completely understood.

Exercise training reduces the incidence of age-related cardiovascular disease [8, 9]. The beneficial effect of exercise on cardioprotection is partially associated with an improvement in mitochondrial oxidative phosphorylation efficiency, an upregulation of mitochondrial biogenesis, an increase in the levels of ROS-scavenging enzymes and other stress response genes, and a reduction in the accumulation of oxidative damage [10, 11]. The cardioprotective effects of exercise also increase the levels of a number of classical preconditioning molecules, such as catalase, heat shock proteins (HSPs), and mitochondrial ATP-sensitive potassium (_*m*_K_ATP_) channels [12]. However, the precise mechanisms responsible for this cardioprotective effect of exercise in restoring PC protection in the aged heart are currently unknown.

Polyamines, such as putrescine (Pu), spermidine (Spd), and spermine (Sp), are organic cations that are required for cell growth; cell differentiation; and the synthesis of DNA, RNA, and proteins [13]. Polyamines have various functions, including the modulation of membrane receptor complexes and several intracellular signal transduction pathways [14–16]. The reaction catalyzed by ornithine decarboxylase (ODC) is the first rate-limiting step in the polyamine biosynthetic pathway, and spermidine/spermine acetyltransferase (SSAT) is another key regulatory enzyme in the catabolism of polyamines. SSAT catalyzes the acetylation of Spd or Sp to produce ROS and toxic aldehydes [17]. Polyamine levels decrease during the aging process in mammals [18, 19]. The rapid activation of ODC and an increase in polyamines are a characteristic cellular response to various stress stimuli, such as heat stress [20], exercise training [21, 22], irradiation [23], and ischemia [24]. Polyamines function as ROS scavengers and are anti-inflammatory [25–28]. Previously, we demonstrated that the upregulation of polyamine biosynthesis is an important step in PC-induced cardioprotection in the adult rat heart [29]. However, to our knowledge, no evidence is available concerning the role of exercise-related polyamine metabolism in restoring preconditioning protection in the aged heart.

In the present study, we first evaluated the effect of IPC on polyamine metabolism in isolated aged rat hearts. Next, we examined whether polyamine metabolism was altered by exercise and whether this change inhibited the loss of preconditioning protection in aged hearts. Furthermore, we focused on the mitochondrial mechanism and the role that polyamines played during this process.

## 2. Materials and Methods

### 2.1. Materials

D,L-*α*-Difluoromethylornithine (DFMO), collagenase type I, and all other chemicals were obtained from Sigma Chemical Co. (St. Louis, MO, USA) unless otherwise indicated. The primary antibodies anti-HSP70, anti-cyclooxygenase-2 (COX-2), anti-cytochrome c (Cyt c), and anti-VDAC were obtained from Santa Cruz Biotechnology (CA, USA). DCFH-DA and JC-1 were obtained from Molecular Probes (Carlsbad, CA, USA).

### 2.2. Animals

The male Wistar rats aged 3 months (young) and 18 months (old) were obtained from the Animal Center of Harbin Medical University. They were treated in accordance with the Guide for Care and Use of Laboratory Animals published by the China National Institutes of Health. All animals were housed under conditions of constant temperature and humidity, a standard light/dark cycle (12 h/12 h), and free access to standard rodent chow and water.

### 2.3. Exercise Training Protocol

Old rats were subjected to treadmill exercise for 6 weeks. They were initially made to run at 20 m/min for 15 min/day and 5 days/week. The speed and duration were gradually increased until the rats were running at 30 m/min for 60 min/day and 5 days/week. This final intensity was maintained for 6 weeks. After the last exercise training session, the rats were allowed to rest for 24 h before the perfused heart experiment was conducted.

### 2.4. Isolated Rat Heart Preparation

For all protocols, the rats were anesthetized with sodium pentobarbital (60 mg/kg body weight, i.p.) and anticoagulated with heparin (1000 IU/kg, i.p.). The hearts were removed and immediately perfused through the aorta at a constant pressure of 100 cm H_2_O using KH buffer (118.50 mM NaCl, 25.00 mM NaHCO_3_, 4.75 mM KCl, 1.19 mM MgSO_4_·7H_2_O, 1.18 mM KH_2_PO_4_, 1.36 mM CaCl_2_·2H_2_O, and 11.10 mM glucose) at 37.0°C and saturated with a gas mixture of O_2_-CO_2_ (95-5%) (pH 7.4).

### 2.5. Protocol 1

**Figure 1:**
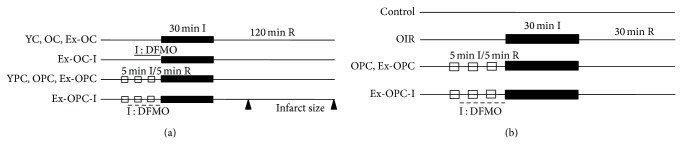
Experimental protocol 1 (a) was used to subject young (Y), old (O), and exercised old rat hearts (Ex-O) to ischemia/reperfusion and ischemic preconditioning treatment. Experimental protocol 2 (b) was used to subject old rat hearts (O) to ischemia/reperfusion and ischemic preconditioning treatment.

A total of 32 young and 96 old Wistar rats were used in protocol 1. Half of the young and the old hearts were used to measure heart infarct size, and the others were used to detect hemodynamics, lactate dehydrogenase (LDH) activity, polyamine levels, and ODC and SSAT activity. Isolated rat hearts were preperfused for 15 min to establish equilibrium. Young, old, and exercised old control hearts were subjected to 30 min of ischemia and 30 or 120 min of reperfusion (YC, OC, and Ex-OC groups, *n* = 8, resp.). Additionally, young, old, and exercised old hearts underwent 3 cycles of 5 min of ischemia and 5 min of reperfusion, followed by 30 min of global ischemia and 30 or 120 min of reperfusion (YPC, OPC, and Ex-OPC groups, *n* = 8, resp.). The hearts of the Ex-OC-I and Ex-OPC-I groups were treated similarly to those of the Ex-OC and Ex-OPC groups, except that the hearts received 2 mM DFMO (an ODC inhibitor) for 15 min before the prolonged ischemia or during the PC cycles before the global ischemia (*n* = 8, resp.) (Figure 1(a)).

#### 2.5.1. Determination of Infarct Size and LDH Activity

At 120 min after reperfusion, the hearts were frozen and cut into 2-mm-thick slices. The slices were thawed and incubated in a 1% tetrazolium chloride (TTC) phosphate-buffered solution at 37°C for 20 min and fixed in 10% formalin. Infarct size was expressed as a percentage of the risk zone [30]. The coronary effluent was collected for 2 min during the reperfusion period, and LDH activity in the coronary effluent was determined using an LDH activity detection kit (NJBI, Nanjing, China).

#### 2.5.2. Hemodynamic Measurements

At the beginning of the experiment, a water-filled balloon connected to a pressure transducer (Chart 5.0 software; AD Instruments Inc., Australia) was inserted into the left ventricle and inflated to an end-diastolic pressure of 7.0 ± 0.5 mmHg. The volume of the balloon was kept constant throughout the experiment to assess cardiodynamic function. The left ventricular developed pressure (LVDP, mmHg), left ventricular end-diastolic pressure (LVEDP, mmHg), and maximal positive and negative change in the left ventricular pressure (±*dp*/*dt*, mmHg/s) were monitored and recorded at 5-min intervals throughout the perfusion period, and the rate pressure product [RPP = LVDP × heart rate] was calculated.

#### 2.5.3. Electron Microscopy Imaging

After 30 min of reperfusion, a portion of the left ventricle was promptly dissected and fixed in glutaraldehyde for 2–4 h at 4°C. The specimen was washed, dehydrated, embedded in resin, and sectioned. The sections were stained with uranyl acetate and lead nitrate and then visualized with an electron microscope (S4800 Hitachi, Tokyo, Japan).

#### 2.5.4. Determination of Polyamine Content and ODC and SSAT Activities

The polyamine content in the cardiac tissue was measured by high-performance liquid chromatography (Waters Co., Milford, Massachusetts, USA) as described in our previous study [29]. ODC activity was measured by determining the amount of ^14^CO_2_ released from L-[1-^14^C] ornithine, and the radioactivity was measured using a liquid scintillation counter (LS-6500 Beckman, USA). The ODC activity was expressed as nmol CO_2_ released/h/mg protein, and the SSAT activity was expressed as nmol acetyl-spermidine formed/min/mg protein.

### 2.6. Protocol 2

Old (*n* = 30) and young (*n* = 3) Wistar male rats were used in protocol 2. Isolated hearts from old rats were allowed to stabilize for 15 min before perfusion and were then randomly divided into five groups. The old control group (OC, *n* = 6) included isolated hearts that were perfused continuously for 105 min. The OIR group (*n* = 6) included hearts that were subjected to 30 min of global ischemia, followed by 30 min of reperfusion. The OPC group (*n* = 6) included hearts that were subjected to 3 cycles of 5 min ischemia/5 min reperfusion, followed by 30 min of global ischemia and 30 min of reperfusion. The Ex-OPC group (*n* = 6) included hearts from exercised rats that were treated the same as those described in the OPC group. In the Ex-OPC-I group (*n* = 6), the procedure was similar to that in the Ex-OPC group, except that 2 mM DFMO was infused throughout the PC period (Figure 1(b)). In addition, we examined the effect of spermine and spermidine on state 3 respiration in mitochondria isolated from three young rat hearts.

#### 2.6.1. Isolation of Cardiac Mitochondria

Mitochondria were isolated at 4°C by differential centrifugation using a mitochondria isolation kit (Baosai Biosciences Inc., Beijing, China). The final mitochondrial pellet was resuspended in isolation buffer, stored on ice, and used for experiments within 4 h. The protein concentration was determined using the Lowry assay kit (Beyotime Inc., Shanghai, China).

#### 2.6.2. Measurement of Mitochondrial Oxygen Consumption, the ADP/O Ratio, and Proton (H^+^) Leakage

Mitochondrial oxygen consumption was measured using a Clark-type oxygen electrode (Hansatech Instruments, Norfolk, UK) at 30°C in mitochondrial respiration buffer (125 mM KCl, 5 mM K_2_HPO_4_, 20 mM MOPS, 2.5 mM EGTA, 1 *μ*M Na_4_P_2_O_7_, and 0.1% bovine serum albumin, pH 7.4) [31]. Pyruvate (5 mM) and malate (5 mM) were used as substrates for complex I-containing mitochondria at a final concentration of 500 *μ*g protein/mL. ADP-stimulated oxygen consumption (state 3 respiration) was measured in the presence of 200 *μ*M ADP, and ADP-independent oxygen consumption (state 4 respiration) was also monitored. The respiratory control ratio (RCR, state 3 divided by state 4) reflects oxygen consumption by phosphorylation (coupling). The ADP/O ratio (number of ADP molecules added for each oxygen atom consumed) is an index of the efficiency of oxidative phosphorylation. Proton leakage was measured in the absence of ATP phosphorylation respiration and in the presence of oligomycin A (1.67 *μ*M). Oligomycin A was added to the reaction chamber to titrate respiration.

#### 2.6.3. Mitochondrial Swelling Analysis

Opening of the mitochondrial permeability transition pore (mPTP) was determined by calculating the Ca^2+^-induced swelling of isolated mitochondria, which was measured as the reduction in absorbance at 520 nm (A520), as previously described [32] with minor modifications. The isolated mitochondria (0.5 mg/mL) were resuspended in swelling buffer (120 mM KCl, 5 mM KH_2_PO_4_, 50 mM MOPS, pH 7.4) with pyruvate (5 mM) and malate (5 mM) as substrates. The mitochondria were then treated with 200 *μ*M CaCl_2_. The decrease in absorbance was monitored at 1-min intervals for 20 min using a spectrophotometer.

#### 2.6.4. Western Blot Analysis of Mitochondrial Protein Expression in Rat Cardiac Tissues

Mitochondria isolated from the left ventricle were subjected to sodium dodecyl sulfate-polyacrylamide gel electrophoresis and blotted on polyvinylidene fluoride membranes (Millipore, Billerica, MA, USA). The membranes were incubated with antibodies against Cyt c, HSP70, COX-2, and VDAC. The secondary antibody was AP-IgG (Promega, Madison, WI, USA). The density of each protein band was quantified using a Bio-Rad ChemiDoc EQ densitometer and Bio-Rad Quantity One software (Bio-Rad Laboratories, Hercules, CA, USA). The protein concentration was quantified using the BCA Protein Assay kit (Beyotime, Nantong, China).

### 2.7. Protocol 3

*Preparation of Isolated Rat Ventricular Cardiomyocytes and Grouping*. Three young and six old male rats were used in protocol 3. Cardiomyocytes from young (Y, *n* = 6), old (O, *n* = 6), and exercised old (old rats subjected to gradient treadmill exercise for 6 weeks, Ex-O, *n* = 6) rats were isolated by enzymatic dissociation with 0.5 mg/mL collagenase type I as previously described [30]. The isolated rat ventricular cardiomyocytes were assigned to different groups as follows: (1) in the control groups (YC, OC, and Ex-OC), the myocytes were only perfused with normoxic Tyrode's solution for 180 min; (2) in the ischemia and reperfusion mimic groups (YIR, OIR, and Ex-OIR), after the cells were preincubated with normoxic Tyrode's solution for 10 min, they were exposed to a hypoxic solution for 90 min and then subjected to normoxic perfusion for 60 min; (3) in the preconditioned groups (YPC, OPC, and Ex-OPC), preconditioning was created with 1 cycle of 10 min of hypoxia and 10 min of reperfusion before the mimic IR; and (4) in the DFMO group (Ex-OPC-I), the procedure was similar to that in the Ex-OPC group, except that 2 mM DFMO was infused throughout preconditioning. Normoxic Tyrode's solution contained 132 mM NaCl, 10 mM HEPES, 5 mM D-glucose, 4.8 mM KCl, 1 mM CaCl_2_, and 1.2 mM MgCl_2_ (pH 7.4) and was maintained at 98% O_2_ and 2% N_2_. Hypoxia was produced by superfusing the myocytes with glucose-free Tyrode's solution (132 mM NaCl, 10 mM HEPES, 8 mM KCl, 1 mM CaCl_2_, 1.2 mM MgCl_2_, 1 mM Na_2_HPO_4_, and 10 mM lactate, pH 6.8) and gassing them with 100% N_2_ in a hypoxic chamber. All experiments using cardiomyocytes were performed at room temperature.

#### 2.7.1. Monitoring of ROS Production in Cardiomyocytes

The fluorescent probe 2′,7′-dichlorodihydrofluorescein diacetate (DCFH-DA) was used to detect the presence of intracellular ROS. The isolated cardiomyocytes were washed with phosphate-buffered saline (PBS) and incubated with 10 *μ*M DCFH-DA according to the manufacturer's protocol. After a 30-min incubation period in the dark, the cells were washed again with PBS. Intracellular DCFH-DA was rapidly oxidized to the fluorescent compound 2′,7′-dichlorofluorescein (DCF) in the presence of ROS. The fluorescence of DCF was measured using excitation and emission wavelengths of 480 and 535 nm, respectively. Thirty intact individual cardiomyocytes in each group were randomly photographed, and their total fluorescence was recorded.

#### 2.7.2. Monitoring of Mitochondrial Membrane Potential (ΔΨ*m*) in Cardiomyocytes

Rat ventricular cardiomyocytes were incubated with tetramethylrhodamine ethyl ester (JC-1, 1 *μ*g/mL) for 30 min at room temperature according to the manufacturer's protocol and were then observed immediately by fluorescence microscopy (BX51 M; Olympus, Japan). Thirty intact individual cardiomyocytes in each group were randomly photographed. Changes in the mitochondrial ΔΨ*m* were assessed by comparing the ratios of the optical density at 590–600 nm (red) to the optical density at 527–534 nm (green). The ratio of the intensity of the JC-1 aggregate (red) to that of the monomer (green) was calculated.

### 2.8. Statistical Analysis

Data from the experiments are expressed as the mean ± standard error (SE). One-way analysis of variance was used for the statistical evaluation of differences among groups. Student's *t*-tests of unpaired data were used when appropriate. A *P* value of less than 0.05 was considered to be statistically significant.

## 3. Results

### 3.1. Effect of Aged and Exercise on Heart and Body Weight in Rats

The mean heart and body weight measurements are shown in Table 1. Heart weight (HW), body weight (BW), and the ratio of the heart weight to body weight (HW/BW) were significantly greater in the old rats than in the young rats. Compared with the old rats, exercise enhanced the HW and the HW/BW ratio and decreased the BW, which confirmed the efficacy of the exercise training regimen used.

### 3.2. Effect of Aging and Exercise on Infarct Size and LDH Activity in Old PC Hearts

**Figure 2:**
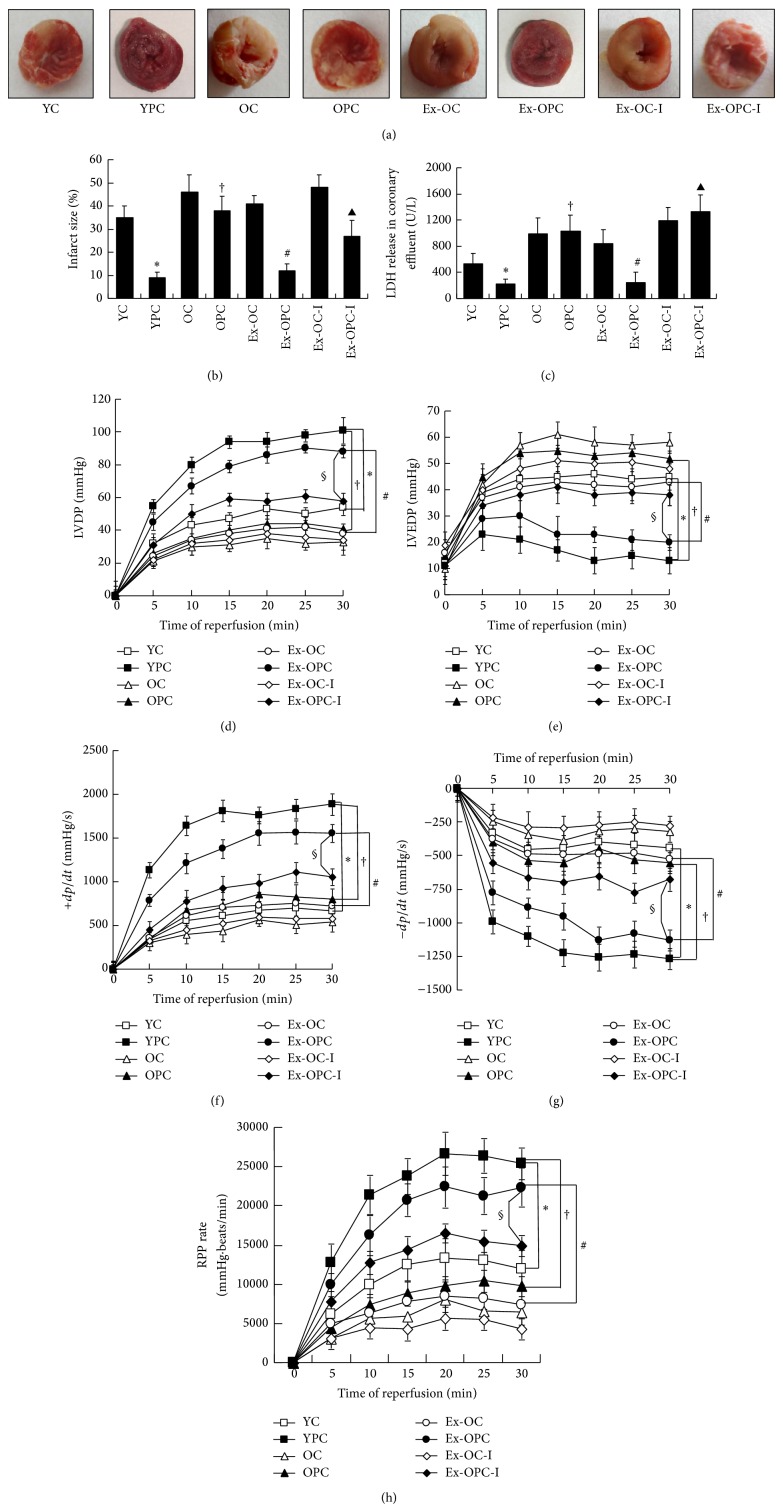
(a) Infarct size was measured using tetrazolium chloride (TTC) staining to distinguish necrotic from viable myocardium. (b) Infarct size (expressed as the percentage of risk zone of the total left ventricle [LV]). (c) Lactate dehydrogenase (LDH) released from the coronary effluent in the different groups. The data of infarct size and LDH are presented as the mean ± SE (*n* = 8 per group). ^*^ *P* < 0.05 versus the YC group; ^†^ *P* < 0.05 versus the YPC group; ^#^ *P* < 0.05 versus the Ex-OC group; ^▲^ *P* < 0.05 versus the Ex-OPC group. ((d)–(h)) The changes in various hemodynamic parameters following reperfusion for 30 min are shown for the young IR control group (YC), young preconditioning group (YPC), old IR control group (OC), old preconditioning group (OPC), exercised old IR control group (Ex-OC), exercised old preconditioning group (Ex-OPC), and DFMO treatment groups (DFMO was administered to the Ex-OC and Ex-OPC groups, resp., presented as the Ex-OC-I and the Ex-OPC-I). Data are presented as the mean ± SE (*n* = 8 per group). ^*^ *P* < 0.05 versus the YC group; ^†^ *P* < 0.05 versus the YPC group; ^#^ *P* < 0.05 versus the Ex-OC group; ^§^ *P* < 0.05 versus Ex-OPC group. LVDP, left ventricular developed pressure; LVEDP, left ventricular end-diastolic pressure; ±*dP*/*dt*, maximal positive and negative change in the left ventricular pressure; RPP, rate pressure product.

In young rats, the preconditioning treatment significantly reduced infarct size and LDH release compared with the IR control group [9.6 ± 3.7% versus 35.8 ± 4.2% (YC) of the infarct size and 219.5 ± 79.1 versus 527.1 ± 167.3 U/L (YC) of the LDH activity, respectively, *P* < 0.05 for all]. In contrast, there was no significant reduction in infarct size and LDH release in the aged hearts upon PC treatment [38.8 ± 6.5% (OPC) versus 46.1 ± 8.2% (OC) of the infarct size and 1036.5 ± 264.5 (OPC) versus 989.5 ± 245.7 U/L (OC) of the LDH activity, resp., *P* > 0.05 for all]. Compared with the YPC group, the infarct sizes and LDH activity were significantly increased in the OPC groups (OPC versus YPC, *P* < 0.05, resp.). After exercise, the infarct size of 39.8 ± 2.2% and LDH activity of 943 ± 58.2 U/L in the Ex-OC hearts were reduced to 12.8 ± 2.2% and 248 ± 23.6 U/L in old P hearts (Ex-OPC versus OPC, *P* < 0.05, resp.). In contrast, the effects of exercise on infarct size and LDH activity were abolished by DFMO (an inhibitor of ODC). DFMO increased the infarct size to 27.4 ± 3.0% and LDH activity to 1333 ± 287.1 U/L with respect to exercised old PC hearts (versus Ex-OPC, *P* < 0.05 for all) (Figures 2(a), 2(b), and 2(c)).

### 3.3. Effect of Aging and Exercise on Hemodynamic Parameters in Old PC Hearts

No significant differences in baseline hemodynamic parameters (LVDP, LVEDP, ±*dp*/*dt*, and RPP) were observed across age and treatment groups. After 30 min of global ischemia, PC induced a significant increase in the LVDP, +*dp*/*dt*, and RPP and a decrease in the LVEDP and −*dp*/*dt* in the young hearts compared with the control hearts that underwent ischemia and reperfusion (YPC versus YC, *P* < 0.05 for all). However, in the old hearts, there were no significant differences in the LVDP, LVEDP, ±*dp*/*dt*, or RPP between the IR control and preconditioned hearts. Additionally, we observed a significant decrease in the LVDP, +*dp*/*dt*, and RPP and a significant increase in the LVEDP and −*dp*/*dt* in the old PC hearts compared with the YPC hearts (OPC versus YPC, *P* < 0.05 for all). Furthermore, we observed that the increase in the LVDP, +*dp*/*dt*, and RPP and the decrease in the LVEDP and −*dp*/*dt* were more pronounced in the exercised old PC hearts than that in the Ex-OC hearts. In contrast, the administration of DFMO during the short PC cycles abolished the effect of exercise on the LVDP, LVEDP, ±*dp*/*dt*, and RPP (Ex-OPC-I versus Ex-OPC, *P* < 0.05 for all) (Figures 2(d), 2(e), 2(f), 2(g), and 2(h)).

### 3.4. Effect of Aging and Exercise on the Ultrastructure of Old PC Hearts

**Figure 3:**
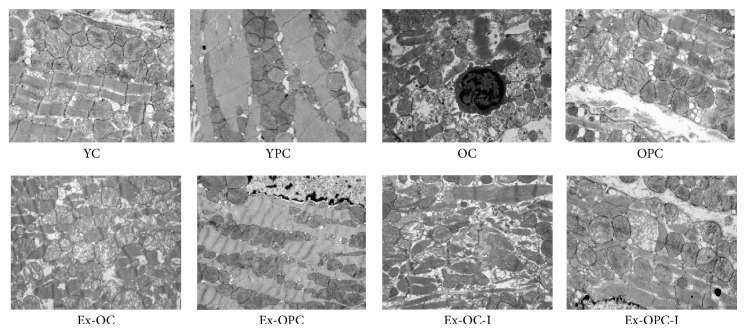
Electron microscopy of the left ventricular ultrastructure. Representative hearts from the young IR control group (YC), young PC group (YPC), old IR control group (OC), old PC group (OPC), exercised old IR group (Ex-OC), exercised old PC group (Ex-OPC), and DFMO treatment groups (Ex-OC-I and Ex-OPC-I groups, DFMO was administered to the Ex-OC and Ex-OPC groups, resp.). *n* = 3 per group. Magnification: ×10000.

In the young IR hearts, we observed ultrastructural abnormalities in the mitochondria, including matrix swelling, cristae disorganization, and inner mitochondrial membrane damage. In the young PC hearts, we observed the mitochondria were tightly packed between the myofibrils and had intact outer and inner membranes with distinct cristae. However, the hearts from the OC, OPC, and Ex-OC groups showed sarcomeric degeneration, some of their mitochondrial cristae were dissolved, and their mitochondrial matrix density was decreased. In contrast, the Ex-OPC hearts showed clear cardiac sarcomeres and a dense mitochondrial matrix with ridges arranged in neat rows. After administering DFMO to Ex-OPC hearts, we observed irregularly arranged myofibrils, ruptured mitochondrial outer membranes, and loose mitochondria matrix (Figure 3).

### 3.5. Effect of Aging and Exercise on Polyamine Metabolism in PC Hearts

We observed the changes in the levels of cardiac polyamine, including Pu, Spd, and Sp, and in the activities of ODC and SSAT (Table 2). In the young rat hearts, we observed that PC caused a significant increase in the ODC activity and tissue Sp and Spd content as well as a decrease in the SSAT activity and Pu levels compared with the IR control group [ODC: 21.40 ± 3.11 versus 16.26 ± 2.11 nmol/mg pro/h (YC); Sp: 212.36 ± 18.72 versus 180.12 ± 22.43 nmol/g wet weight (YC); Spd: 233.94 ± 11.03 versus 189.10 ± 42.03 nmol/g wet weight (YC); SSAT: 4.14 ± 0.58 versus 6.78 ± 0.23 nmol/mg pro/h (YC); Pu: 4.14 ± 1.18 versus 8.08 ± 3.03 nmol/g wet weight (YC), *P* < 0.01 and *P* < 0.05, resp.]. In contrast, in old rat hearts, the activities of ODC and SSAT and the tissue levels of Sp, Spd, and Pu were not significantly different between the OPC and OC hearts [ODC: 12.30 ± 1.89 versus 11.78 ± 2.11 nmol/mg pro/h (OC); SSAT: 9.17 ± 1.23 versus 9.99 ± 0.93 nmol/mg pro/h (OC); Sp: 129.44 ± 29.53 versus 143.59 ± 13.43 nmol/g wet weight (OC); Spd: 106.14 ± 32.38 versus 121.14 ± 15.67 nmol/g wet weight (OC); Pu: 7.69 ± 1.06 versus 9.98 ± 2.08 nmol/g wet weight (OC), resp., *P* > 0.05 for all]. Compared with the YPC hearts, the ODC activity and the tissue levels of Sp and Spd were decreased and the SSAT activity and the tissue level of Pu were significantly increased in the OPC hearts (versus YPC, *P* < 0.01 and *P* < 0.05, resp.). Surprisingly, exercise significantly increased the cardiac ODC activity as well as the Sp and Spd levels and significantly decreased the Pu content and SSAT activity in old PC hearts compared with exercised old IR control hearts (Ex-OC) [ODC: 18.10 ± 1.41 versus 13.34 ± 1.27 nmol/mg pro/h (Ex-OC); Sp: 169.84 ± 14.03 versus 130.41 ± 10.03 nmol/g wet weight (Ex-OC); Spd: 157.91 ± 14.58 versus 123.02 ± 12.2 nmol/g wet weight (Ex-OC); SSAT: 4.08 ± 0.88 versus 7.19 ± 0.69 nmol/mg pro/h (Ex-OC); Pu: 3.89 ± 2.18 versus 7.45 ± 1.53 nmol/g wet weight (Ex-OC), *P* < 0.01 and *P* < 0.05, resp.]. Furthermore, when DFMO was administered over the 5-min PC cycles, the ODC activity and the tissue levels of Sp, Spd, and Pu were decreased to 10.99 ± 1.21, 124.76 ± 15.11, 98.21 ± 14.35, and 3.39 ± 1.31 (Ex-OPC-I versus Ex-OPC, *P* < 0.01 and *P* < 0.05, resp.). These data indicate that ODC/polyamine pathway is most likely involved in the PC phenomenon and that the restoration of exercising induced PC in old hearts may result from the restoration of the cardiac polyamine pool in response to a PC stimulus.

### 3.6. Effect of Aging and Exercise on Mitochondrial Respiration in Old PC Hearts

**Figure 4:**
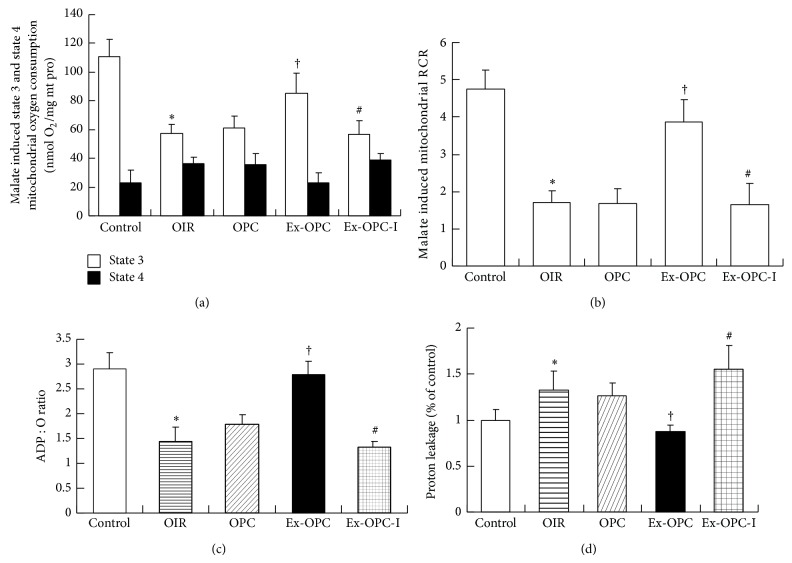
Mitochondrial oxidative phosphorylation efficiency was evaluated based on mitochondrial state 3 and 4 oxygen consumption (a), the respiratory control rate (RCR) (b), the ADP/O ratio (the nanomoles of ADP phosphorylated by the nanomoles of O_2_) (c), and proton leakage (oligomycin-inhibited respiration) (d). Respiration was induced with pyruvate/malate (5 mM each) as energizing substrates and ADP (200 *μ*M) to initiate state 3 respiration. Data are presented as the mean ± SE (*n* = 6 per group). ^*^ *P* < 0.05 versus the control group; ^†^ *P* < 0.05 versus the OPC group; ^#^ *P* < 0.05 versus the Ex-OPC group.

We measured mitochondrial respiratory function, including the respiratory rates of states 3 and 4 (Figure 4(a)), the RCR (Figure 4(b)), the ADP/O ratio (Figure 4(c)), and proton leakage (Figure 4(d)) using pyruvate/malate as substrates. Mitochondrial proton leakage was measured as oligomycin-inhibited state 3 respiration, which was normalized to controls. We observed that the state 3 respiratory rate, RCR, and ADP/O ratio were significantly decreased and the proton leakage was significantly increased in the OIR group compared with the old control group (*P* < 0.05 for all). However, there were no significant differences in any of these indices between the OPC and OC groups. Interestingly, the state 3 respiratory rate, RCR, and ADP/O ratio were higher and the oxidative phosphorylation-independent proton leakage was lower in the exercised old preconditioned hearts than in the OPC hearts (*P* < 0.05 for all). However, these effects were significantly restrained in the DFMO-treated hearts, indicating that inhibiting polyamine synthesis decreased the efficiency of mitochondrial oxidative phosphorylation coupling. However, there were no significant differences in the mitochondrial state 4 respiratory rate among any of the experimental groups.

### 3.7. Effect of Aging and Exercise on the Opening of the mPTP in Old PC Hearts

**Figure 5:**
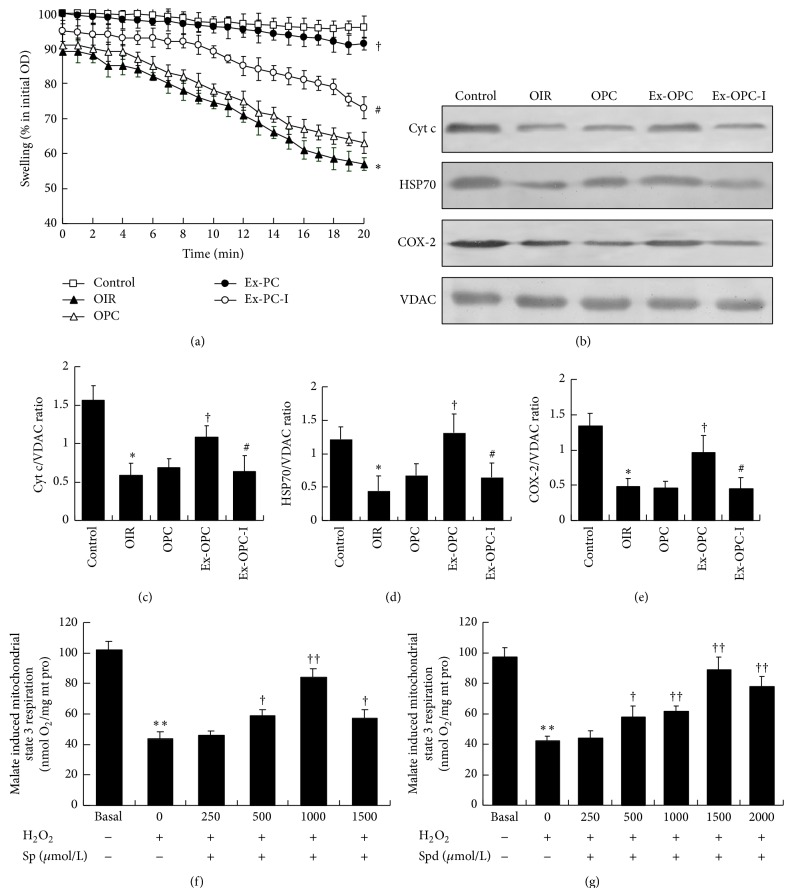
(a) The results from six similar experiments of mitochondrial swelling are shown. Mitochondria (0.5 mg/mL) were incubated in standard incubation medium. After 1.5 min of incubation, the mitochondria were loaded with 200 *μ*M CaCl_2_. Data are expressed as the mean ± SE. ^*^ *P* < 0.05 versus the control group; ^†^ *P* < 0.05 versus the OPC group; ^#^ *P* < 0.05 versus the Ex-OPC group. (b) Changes in the mitochondrial expression of Cyt c, HSP70, and COX-2 as determined by western blot. A quantitative analysis of the mitochondrial Cyt c (c), HSP70 (d), and COX-2 (e) expression levels is also shown. VDAC was used as an internal control. Data are presented as the mean ± SE (*n* = 4 per group). ^*^ *P* < 0.05 versus the OC group; ^†^ *P* < 0.05 versus the OPC group; ^#^ *P* < 0.05 versus the Ex-OPC group. (f and g) Dose-dependent effect of spermine and spermidine on the state 3 respiratory rate in cardiac mitochondria isolated from young hearts in the presence of 100 *μ*M H_2_O_2_ (*n* = 6-7) for 10 min at 30°C. ^**^ *P* < 0.01 versus basal conditions; ^†^ *P* < 0.05, ^††^ *P* < 0.01 versus H_2_O_2_ treatment.

Mitochondrial swelling was used as an* in vitro* index of the opening susceptibility of the mPTP. We found that calcium (200 *μ*M) stimulation led to mitochondrial swelling, as indicated by a decrease in the A520 (Figure 5(a)). In the old rat hearts, IR caused a significant increase in calcium-induced mitochondrial swelling compared with the control group (*P* < 0.05). PC did not decrease calcium-induced mitochondrial swelling, and the rate and extent of the swelling were similar to those in the IR hearts. The mitochondria in the exercised PC hearts showed substantially decreased swelling compared with the mitochondria in the OPC hearts (*P* < 0.05). Inhibiting ODC with DFMO abolished the effect of exercise on calcium-stimulated mitochondrial swelling.

### 3.8. Effect of Aging and Exercise on the Mitochondrial Protein Expression of Cyt c, HSP70, and COX-2 in Old PC Hearts

In the OC hearts, the expression levels of Cyt c, HSP70, and COX-2 in the mitochondria were relatively high. After IR, the protein expression levels were significantly decreased [Cyt c: 0.59 ± 0.5 versus 1.56 ± 0.19; HSP: 0.44 ± 0.23 versus 1.22 ± 0.18; COX-2: 0.48 ± 0.12 versus 1.34 ± 0.88, resp., all *P* < 0.05]. PC did not affect the expression of Cyt c, HSP70, and COX-2 in the old IR hearts. However, exercise significantly increased the mitochondrial Cyt c, HSP70, and COX-2 expression compared with the OPC group [Cyt c: 1.08 ± 0.12 versus 0.69 ± 0.11; HSP70: 1.31 ± 0.23 versus 0.68 ± 0.13; COX-2: 0.96 ± 0.22 versus 0.46 ± 0.11, resp., all *P* < 0.05]. DFMO administration throughout the PC cycles significantly decreased the protein levels in Ex-OPC hearts; these levels were decreased to 0.64 ± 0.20 (Cyt c), 0.65 ± 0.21 (HSP70), and 0.45 ± 0.16 (COX-2) (versus Ex-OPC, *P* < 0.05 for all) (Figures 5(b), 5(c), 5(d), and 5(e)).

### 3.9. Polyamine-Dependent Changes in State 3 Mitochondrial Oxygen Consumption

To further determine the mechanism by which polyamines elicit mitochondrial protection, we examined the effect of Sp and Spd on state 3 mitochondrial respiration in the presence of 100 *μ*M H_2_O_2_ (Figures 5(f) and 5(g)). We found that the administration of 100 *μ*M H_2_O_2_ to mitochondria isolated from young rat hearts resulted in much lower state 3 respiration than that present under basal conditions (*P* < 0.01). The administration of Sp and Spd 2 min before the administration of H_2_O_2_ increased state 3 respiration rate in a dose-dependent manner. Additionally, 1 mM Sp and 1.5 mM Spd caused a larger increase in the state 3 respiratory rate compared with that observed after treatment with H_2_O_2_ alone (*P* < 0.01 for all).

### 3.10. Effect of Aging and Exercise on ROS Production and ΔΨ*m* in PC Cardiomyocytes

**Figure 6:**
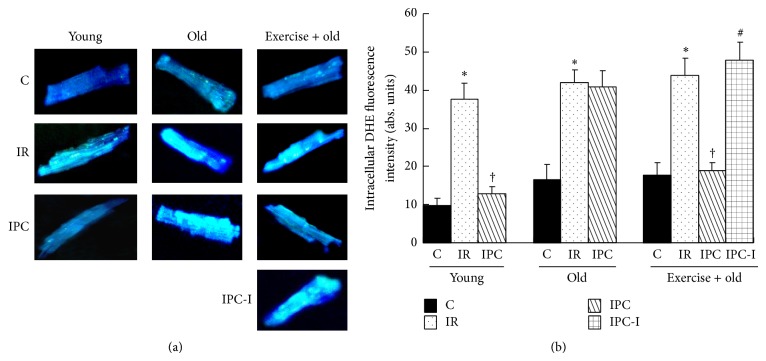
(a) Isolated cardiomyocytes were preloaded with DCFH-DA to measure ROS generation. (b) Statistical analysis of the average fluorescence intensity from the cardiomyocytes. The data shown are the mean ± SE (*n* = 6 per group). ^*^ *P* < 0.05 versus the control group; ^†^ *P* < 0.05 versus the IR group; ^#^ *P* < 0.05 versus exercised old PC group.

**Figure 7:**
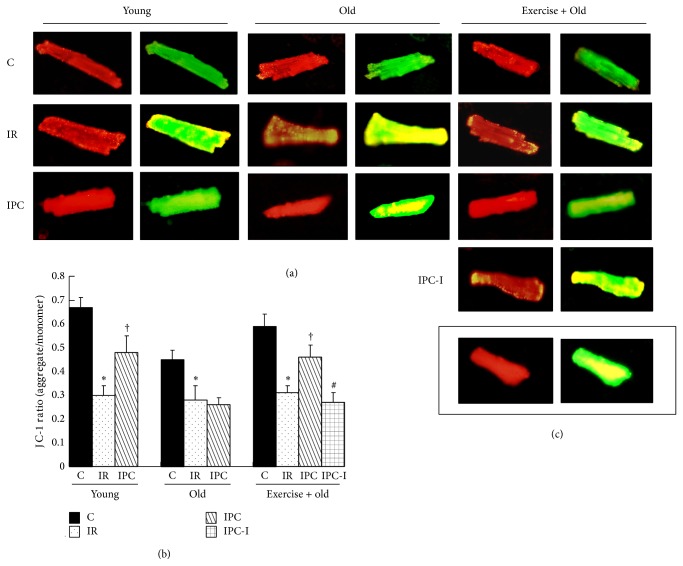
Isolated rat cardiomyocytes were stained with JC-1. (a) The red fluorescence represents the mitochondrial aggregate form of JC-1, indicating an intact ΔΨ*m*. The green fluorescence represents the monomeric form of JC-1, indicating the dissipation of the ΔΨ*m*. (b) Statistical analysis of the ratio of red to green fluorescence. (c) Carbonyl cyanide m-chlorophenyl hydrazone was used as a positive control. The data are shown as the mean ± SE (*n* = 6 per group). ^*^ *P* < 0.05 versus the control group, ^†^ *P* < 0.05 versus the IR group; ^#^ *P* < 0.05 versus exercised old PC group.

ROS levels were measured in isolated cardiomyocytes (Figures 6(a) and 6(b)). JC-1 staining was used to detect the ΔΨ*m* of the isolated cardiomyocytes. The ratio of red and green fluorescence was used to represent the ΔΨ*m* (Figures 7(a) and 7(b)). Carbonyl cyanide m-chlorophenyl hydrazone, the positive control, promoted mitochondrial inner membrane permeability, leading to dissipation of the H^+^ gradient across the inner mitochondrial membrane (Figure 7(c)). We found that IR caused a significant increase in ROS fluorescence and a significant decrease in ΔΨ*m* in the cardiomyocytes from the YIR, OIR, and Ex-OIR groups compared with those from the YC, OC, and Ex-OC groups [ROS: 37.5 ± 4.3 (YIR) versus 9.8 ± 1.7 abs. units (YC); 42.1 ± 2.5 (OIR) versus 16.2 ± 4.6 abs. units (OC); and 43.8 ± 4.2 (Ex-IR) versus 17.3 ± 3.8 abs. units (Ex-C); ΔΨ*m*: 0.31 ± 0.04 (YIR) versus 0.67 ± 0.05 (YC); 0.27 ± 0.06 (OIR) versus 0.46 ± 0.05 (OC); and 0.31 ± 0.06 (Ex-IR) versus 0.61 ± 0.05 (Ex-C), *P* < 0.05 for all]. In contrast, the ROS fluorescence was lower and the ΔΨ*m* was higher in the PC cardiomyocytes isolated from the YPC and Ex-OPC hearts compared with those isolated from the YIR and Ex-OIR hearts [ROS: 11.8 ± 2.2 abs. units (YPC) versus YIR and 18.8 ± 2.0 abs. units (Ex-OPC) versus Ex-OIR; ΔΨ*m*: 0.48 ± 0.02 (YPC) versus YIR and 0.46 ± 0.05 (Ex-OPC) versus Ex-OIR, *P* < 0.05 for all]. However, PC had a weaker influence on ROS levels and ΔΨ*m* compared with the OIR group (OPC versus OIR, *P* > 0.05). Administering DFMO throughout PC cycles abolished the effect of exercise on the ROS fluorescence and ΔΨ*m* in the Ex-OPC hearts. The concentration of ROS was increased to 47.8 ± 4.2 abs. units, and ΔΨ*m* was decreased to 0.27 ± 0.04 (versus Ex-OPC, *P* < 0.05 for all).

## 4. Discussion

Our current study showed that IPC could protect young isolated perfused rat hearts against ischemia/reperfusion (IR) injury; however, IPC protection was ineffective in aged rat hearts and aged isolated cardiomyocytes. Indeed, a range of studies performed in humans and other animals have shown that aged hearts cannot be preconditioned via ischemia [2, 33, 34]. Recent studies indicate that the reduced levels of mitochondrial STAT3 and Cx43 in aged hearts may contribute to the age-related loss of cardioprotection by IPC, and the authors provide evidence that the mitochondria, as an integration point, are critical for cardiomyocyte survival through PC protection [35, 36]. However, a detailed understanding of the molecular mechanisms responsible for this reaction in the aged PC heart is still needed.

Exercise profoundly affects the physiological and pathophysiological modifications induced by aging [37]. Exercise can protect the heart against IR insult and provides cardioprotection in human and animals [12]. We have shown here that exercise inhibited the loss of IPC protection by reducing the infarct size, decreasing the release of LDH, improving the postischemic recovery of heart function, and reducing ultrastructural injury in aged rat hearts. Exercise can also restore the loss of IPC protection in isolated intact aged rat cardiomyocytes. In a separate experiment, we further observed that the cardioprotection of IPC is preserved by exercise in aged rats by increasing the mitochondrial oxidative phosphorylation efficiency, inducing the mitochondrial HSP70 and COX-2 expression, and inhibiting mitochondrial Cyt c release and ROS generation. The results suggest that adaptive changes in the mitochondria may contribute to the exercise-induced cardioprotection in aged PC hearts. Our results are in line with the similar findings by other groups [38, 39].

IPC protection is likely mediated by numerous factors, including adenosine, bradykinin, and opioid receptors. These mediators activate downstream kinases, such as PKC, PI3 K/Akt, and p38 MAPK; these mediators all converge at the mitochondria, which act as an integration point that is critical for cardiomyocyte survival [1–5, 7]. Some studies have confirmed the role of polyamines in controlling the basal level of second messengers and modulating extracellular signal transduction through G protein-coupled receptors, which further activate phospholipase C and subsequently PKC [40]. Recently, it is reported that polyamines modulate some key signal transduction pathways, such as the AMPK, Akt, and p38 MAPK pathways in cardiomyocytes [41]. We have found that activation of PKC-*ε* stimulates ODC expression and polyamine biosynthesis, which play important roles in IPC protection in the adult rat hearts [29]. Accordingly, it is unlikely that the ODC/polyamine system is involved in PC-induced cardiac protection in aged rat hearts.

In the present study, we observed that the ODC activity and tissue Sp and Spd levels were significantly decreased and that the SSAT activity and tissue Pu levels were increased in the old PC hearts compared with the young PC hearts. In contrast, exercise caused an unexpected increase in the Spd and Sp levels and ODC activity as well as a decrease in the SSAT activity in the old PC hearts, and DFMO abolished exercise-mediated cardioprotection in the aged IPC hearts. Thus, we speculate that the reduction in cardiac polyamines may contribute to the impairment of IPC cardioprotection in elderly rats and that exercise restores this protection, most likely by promoting polyamine synthesis, inhibiting polyamine degradation, and further restoring the cardiac polyamine pool.

Intracellular polyamine levels are tightly controlled by biosynthetic and catabolic pathways [14–17], imbalanced polyamine homeostasis is associated with various pathological conditions [18, 19, 29, 42, 43], and exogenous polyamines may be powerful weapons against these deleterious consequences [25–29]. Our prior work suggests that exogenous spermine confers cardioprotective effects against IR by stabilizing the cell membrane, scavenging free radicals, and preventing an increase in intracellular free calcium [24]. Therefore, here the upregulation of ODC activity and the rise in polyamine levels stimulated by exercise may induce antioxidation, increase antiapoptosis, and modify the intracellular calcium levels, which may contribute to the restoration of IPC protection in aged rat hearts. In addition, exercise training decreased the activity of SSAT in aged PC hearts and may result in a decrease in the production of ROS and 3-aminopropanal, both of which result in toxicity and cell death [17]. In particular, DFMO abolished the effect of exercise on aged PC hearts, providing more important evidence that restoration of the intracellular polyamine pool by exercise may be involved in restoring PC protection in aged hearts.

The mitochondria are involved in the process of aging through enhancing the production of mitochondrial ROS and opening of the mPT pore, both of which are important players in cell death [6, 7]. Polyamines act as inhibitors of the mPT [44] and modulate mitochondrial calcium transport [45]. Polyamines are powerful scavengers of hydroxyl radicals and singlet oxygen [26–28], and Spd reduces age-related oxidative damage in mice and increases resistance to H_2_O_2_ [18]. In the present study, we were surprised to find that DFMO decreased the mitochondrial oxidative phosphorylation efficiency, induced mitochondrial swelling, downregulated the mitochondrial protein expression of HSP70 and COX-2, and promoted mitochondrial Cyt c release and ROS generation in the aged PC hearts. In contrast, the administration of exogenous Sp or Spd to the isolated mitochondria before administering H_2_O_2_ significantly improved mitochondrial state 3 respiration in a concentration-dependent manner. These results strongly indicate that there may be a causal relationship between mitochondrial dysfunction and myocardial polyamine depletion in old PC hearts.

Aged hearts have a functional disadvantage in the activation of ODC and lower tissue polyamine concentrations compared with young hearts [18, 19], and the shifts are associated with cardiomyopathy [24, 29]. Conversely, polyamine intake inhibits age-associated cardiovascular pathologies [46, 47]. Thus, our present findings, coupled with the evidence for alterations in polyamine contents with aging, reveal the need for further research into this potentially complex interaction among polyamines, aging, and the reduction in ischemic tolerance and PC cardioprotection. Further elucidation of these mechanisms may aid in the development of therapeutics to restore IPC protection in the aged heart.

Some studies have shown a rapid and significant elevation in ODC activity and polyamine levels during various stressful conditions, including exercise [21, 22]. Here, we confirmed that exercising reestablishes PC protection in old rat hearts through upregulation of the ODC/polyamine system and improvement of mitochondrial function. Obviously, a very complex cascade of events simultaneously acts in the old PC heart in response to exercise, but a detailed mechanism has not been examined in this research, and further studies were needed in this respect.

In conclusion, our study shows for the first time that a loss in the efficacy of preconditioning in aged rat hearts is accompanied by an impaired ability to maintain intracellular polyamine homeostasis and mitochondrial functional integrity. Exercise training appears to restore preconditioning protection in aged rat hearts. This protection appears to be, at least in part, due to an increase in the cardiac polyamine pool and an improvement in mitochondrial function in response to a PC stimulus.

## Figures and Tables

**Table 1:** The effect of age and exercise on heart and body weight in rats.

Rats	HW (mg)	BW (g)	HW/BW (mg/g)
Young (*n* = 10)	730 ± 60	213 ± 31	3.55 ± 0.23
Old (*n* = 9)	1588 ± 155∗	583 ± 25∗	2.98 ± 0.26∗
Exercised old (*n* = 8)	1841 ± 143^†^	484 ± 29^†^	3.87 ± 0.25^†^

Values are means ± SE. BW, body weight; HW, heart weight.

^*^ *P* < 0.05 versus young rats; ^†^ *P* < 0.05 versus old rats.

**Table 2:** Changes in ODC and SSAT enzyme activity and polyamine levels in rat cardiac tissue.

Groups	ODC activity	SSAT activity	Putrescine	Spermidine	Spermine
	nmol CO_2_ released/h/mg pro	nmol acetyl-spd formed/min/mg pro	nmol/g tissue wet weight		
YC	16.25 ± 2.11	6.89 ± 1.18	8.03 ± 2.84	189.13 ± 13.80	186.72 ± 10.27
YPC	21.54 ± 2.65^**^	4.13 ± 0.57^**^	4.56 ± 1.99^*^	233.90 ± 17.55^**^	208.06 ± 15.19^*^
OC	11.94 ± 1.82	9.99 ± 0.97	9.97 ± 2.02	121.14 ± 16.67	148.59 ± 14.76
OPC	12.31 ± 1.63^††^	9.17 ± 1.20^††^	7.69 ± 1.98^†^	108.14 ± 15.77^††^	132.44 ± 13.85^††^
Ex-OC	13.01 ± 1.38	7.08 ± 0.86	7.25 ± 1.97	129.87 ± 13.73	130.03 ± 11.61
Ex-OPC	18.09 ± 1.61^##^	4.09 ± 0.98^##^	4.07 ± 1.76^#^	156.03 ± 16.85^#^	169.81 ± 15.46^##^
Ex-OC-I	10.86 ± 1.19^#^	6.58 ± 0.69	4.01 ± 1.84^#^	87.76 ± 16.61^##^	110.58 ± 13.76^#^
Ex-OPC-I	10.91 ± 1.25^§§^	5.11 ± 0.88	2.33 ± 0.53^§^	98.21 ± 15.82^§§^	124.71 ± 15.75^§§^

Treatment groups are YC, OC and Ex-OC groups, isolated perfused young, old, and old exercised rat heart with IR treatment; YPC, OPC and Ex-OPC groups, isolated perfused young, old, and old exercised rat heart with IPC treatment; and Ex-OC-I and Ex-OPC-I groups, ODC inhibitor DFMO was given to both the Ex-OC and Ex-OPC heart before prolonged ischemia, respectively. Data are the mean ± SE, *n* = 8 per group. ^*^ *P* < 0.05, ^**^ *P* < 0.01 versus the YC group; ^†^ *P* < 0.05, ^††^ *P* < 0.01 versus the YPC group; ^#^ *P* < 0.05, ^##^ *P* < 0.01 versus the Ex-OC group; ^§^ *P* < 0.05, ^§§^ *P* < 0.01 versus the Ex-OPC group.

## Abbreviations

PC: Preconditioning
IR: Ischemia/reperfusion
ROS: Reactive oxygen species
Ex: Exercise
HSPs: Heat shock proteins
KATPm: Mitochondrial ATP-sensitive potassium
Pu: Putrescine
Spd: Spermidine
Sp: Spermine
ODC: Ornithine decarboxylase
SSAT: Spermidine/spermine acetyltransferase
DFMO: D, L-*α*-Difluoromethylornithine
COX-2: Cyclooxygenase-2
Cyt c: Cytochrome c
TTC: Tetrazolium chloride
LDH: Lactate dehydrogenase
LVDP: Left ventricular developed pressure
LVEDP: Left ventricular end-diastolic pressure
±dP/dt: Maximal positive and negative change in the left ventricular pressure
RPP: Rate pressure product
RCR: Respiratory control ratio
mPTP: Mitochondrial permeability transition pore
O: Old
Y: Young
DCFH-DA: 2′,7′-Dichlorodihydrofluorescein diacetate
JC-1: Tetramethylrhodamine ethyl ester
CCCP: Carbonyl cyanide m-chlorophenyl hydrazone
STAT3: Signal transducers and activator of transcription 3
AKT: Phosphorylated protein kinase B.
